# Long term use of lokivetmab (Cytopoint®) in atopic dogs

**DOI:** 10.1186/s12917-025-04645-8

**Published:** 2025-03-26

**Authors:** Margaret Gober, Deb Amodie, Marnie Mellencamp, Andrew Hillier

**Affiliations:** Zoetis LLC, 10 Sylvan Way, NJ Parsippany, 07054 USA

**Keywords:** Cytopoint, Lokivetmab, Dogs, Canine atopic dermatitis, Allergic dermatitis

## Abstract

Lokivetmab (Cytopoint®, Zoetis) has been shown to be effective for the short-term treatment of dogs with allergic and atopic dermatitis but there are no studies at US label dosing (at least 2 mg/kg every 4–8 weeks as necessary) which evaluate long-term usage. The objective of this study was to follow a cohort of dogs receiving lokivetmab to treat their canine atopic dermatitis (CAD) over 12 months. The initial phase of this interventional cohort study evaluated a dog’s pruritus following monthly injections (up to 3 injections) of lokivetmab. Dogs who achieved pruritus < 36 mm using a Pet Owner Pruritus Visual Analogue Scale (PVAS) scoring system during the initial phase, were included in this study. Dogs received lokivetmab injections per the US label every 4–8 weeks and returned on days 180 and 365 (± 7 days) after their initial Day 0 for examination by investigators. Pet owners were asked to complete an electronic PVAS assessment every 2 weeks. At each visit, investigators completed a Canine Atopic Dermatitis Extent and Severity Index (CADESI-4) score and VetVAS to measure skin lesion scores. There were 87% (64/75) of dogs who maintained a PVAS below their baseline PVAS on Day 0. Over the course of the study, 88% (65/75) of dogs obtained a mean PVAS below 36 mm. Of those dogs, 31% (23/75) achieved a biweekly PVAS that was below 36 for the entirety of the study with 11% (8/75) having a biweekly PVAS score that stayed below 20 (considered normal dog level of pruritus) for the entire study. Most owners (93%; 64/49) were satisfied with lokivetmab with 88% planning to continue lokivetmab usage. The majority (80%; 55/69) of pet owners reported they were able to reduce the use of other products while their dog was using lokivetmab, and 87% (60/69) of owners found caring for their dog’s atopic dermatitis was easier with lokivetmab compared to prior treatments.

## Introduction

Canine atopic dermatitis (CAD) is a common skin disease in dogs [12, 15], and is a hereditary, typically pruritic and predominantly T-cell driven inflammatory skin disease involving interplay between skin barrier abnormalities, allergen sensitization and microbial dysbiosis, [5] with characteristic clinical features, the most prominent of which is persistent pruritus. CAD is estimated to affect 10–15% of the total canine population [12], with higher incidence in certain breeds such as Labrador retrievers, Golden retrievers, spaniels, and terriers [8, 13]. Many dogs with this condition require life-long therapy to manage their clinical signs and to maintain an acceptable quality of life [26].

Pruritus, a characteristic feature of CAD, can have a significant impact on the quality of life of both pet and owner [14]. Atopic dermatitis requires ongoing and often lifelong management [8, 20, 26].

In CAD, a likely defective skin barrier allows allergens (allergenic proteins) to penetrate and initiate abnormal immunological reactions [20]. These reactions involve many different cytokines, including interleukin-31 (IL-31) which has been found to be one of the key pruritogens in several species including dogs [10]. Interleukin-31 binds to receptors in peripheral sensory neurons likely activating pruritogenic signals in peripheral neurons that transmit the itch signal from the skin to the brain [2, 27]. New research provides even more information as to the role IL-31 plays in T_H_2-weighted inflammation through release of various proinflammatory mediators [17]. Once bound, IL-31 maintains ongoing inflammation, modulation of the immune response, and cell differentiation [1]. In addition, IL-31 in humans has been shown to stimulate the secretion of other proinflammatory cytokines (IL-4, IL-6, IL-8, IL-13, IL-16, IL32) as well as chemokines and matrix metalloproteinases from other tissues, which suggests IL-31 plays an active role in chronic inflammation [24, 36].

Lokivetmab (Cytopoint®) has been shown to be effective for the treatment of dogs against allergic dermatitis and CAD [19, 34]. Lokivetmab contains a caninized monoclonal antibody (mAb) against IL-31 [18]. Lokivetmab remains in circulation for several weeks (half-life of 16 days) and exerts a therapeutic effect by binding to and neutralizing soluble IL-31, thus inhibiting pruritus and reducing skin lesions [7]. Field studies demonstrate the efficacy of lokivetmab in reducing pruritus and skin lesion scores of CAD for 4–8 weeks following a single injection [18].

Lokivetmab, a monoclonal antibody treatment for canine allergic and atopic dermatitis, has been shown to have an efficacy comparable to cyclosporin [19] but less secondary adverse effects have been reported with lokivetmab compared to cyclosporin [19]. Based on the lokivetmab safety profile, it is possible a similar reduction in adverse events might occur when comparing the use of lokivetmab to glucocorticoids [8]. Additional proposed advantages of lokivetmab therapy compared to other pruritus treatments include a rapid effect, less frequent dosing, no age restriction, increased compliance, lower caregiver burden, improved animal and owner quality of life and compatibility with other medications [28, 30, 32].

**Fig. 1:**
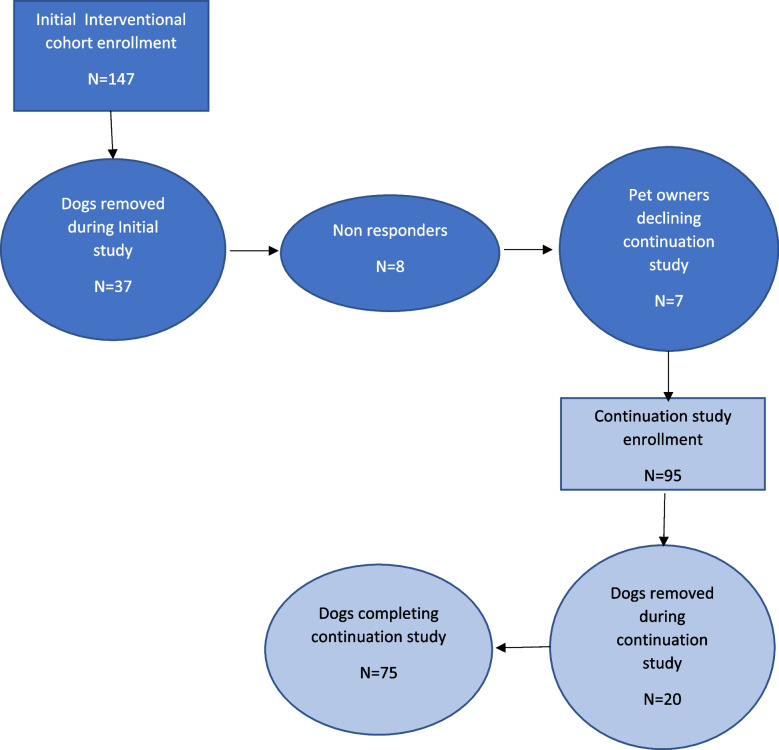
Study enrollment

The initial phase of this interventional cohort study evaluated dog’s pruritus following monthly injections (up to a maximum of 3 monthly injections) of lokivetmab [9]. In the initial phase, dogs with a preponderance of historical data, clinical signs, and diagnostic testing which indicated a diagnosis of atopic dermatitis, confirmed history of non-seasonal dermatitis or seasonal dermatitis of at least 4 months duration and a Pet Owner Pruritus Visual Analogue Scale (PVAS) score < 50 mm were enrolled by dermatologists in referral practice (*n* = 147). Each dog received monthly injections of lokivetmab at a dose of 2 mg/kg. Dogs were reassessed monthly for up to 3 visits (Day 30, 60 and 90). If dogs achieved a reduction in pruritus < 36 mm using the PVAS scoring system at any of the monthly visits, they were offered the opportunity to continue in the study reported herein. A total of 45 dogs were removed during the initial phase of the study including 8 dogs not achieving the primary variable [9]. An additional 7 pet owners declined enrollment in the continuation study, thus a total of 52 dogs were not moved into this continuation study (Fig. 1). The selection of PVAS reduction < 36 as the primary study outcome was selected based on the available COSCAD’18 therapeutic clinical trial recommendations [22] at the time of study initiation.

The objective of this continuation study was to follow a cohort of dogs who initially achieved treatment success with lokivetmab for an additional 9–11 months (resulting in a total of 12 months) and characterize the ongoing impact of lokivetmab in treating their CAD.

## Materials and methods

### Ethics

This study was reported using STROBE guidelines [35]. All study participants were informed of the purpose of this study and written informed consent was obtained. The protocol was reviewed and approved by the Zoetis Ethics Review Board.

### Patient selection

This was a prospective, open interventional study. Investigators were board-certified dermatologists from 8 dermatology specialty practices across the United States. No more than 25% of patient recruitment from a single clinic was allowed. Data was collected from Sept. 1, 2017 to April 15, 2019. Dogs included in this study achieved a reduction of pruritus consistent with a PVAS < 36 mm in an initial 3-month study after receiving 1, or 2 or 3 monthly lokivetmab injections. Each dog had a preponderance of historical data, clinical signs, and diagnostic testing indicating a diagnosis of atopic dermatitis and adverse food reactions, parasitic infestations, fungal or bacterial dermatitis and other metabolic disease had been eliminated as the primary cause of the dog’s clinical signs. Once entered into this continuation study, investigators were instructed to have dogs return on days 180 and 365 (± 7 days) after the initial lokivetmab injection (Day 0) for examination.

### Injection protocol and follow-up evaluation

During this study, investigators were instructed to administer lokivetmab injections per the product label dose of 2 mg/kg, with dogs receiving an injection every 4–8 weeks based on the pet owner’s assessment of pruritus. The additional injections were administered by a technician and did not involve reexamination by the investigator unless deemed necessary by the pet owner, or unless it was a day 180 or day 365 visit. If dogs were using corticosteroid-free and antihistamine-free topical products prior to starting the study, they were permitted to continue using these products, with a maximal frequency for use of wipes, sprays or lotions of every other day and weekly use for shampoo/conditioners. Dogs were also permitted to continue use of oral essential fatty acids and probiotics. If dogs experienced an allergic flare during the continuation portion of the study, they were examined by the investigator and were permitted to receive a “rescue” therapy which could include oclacitinib tablets (Apoquel®, Zoetis), topical anti-inflammatory therapy, antihistamines, glucocorticoids, or other short-term treatment to control the flare as appropriate. If the allergy flare occurred within 4 weeks of a lokivetmab injection, investigators were to administer additional medication until the next lokivetmab injection at 4 weeks and within 3 days thereafter discontinue the “rescue” medication. If an allergy flare occurred 4 −8 weeks after a lokivetmab injection, investigators were to administer lokivetmab again, even if this would be earlier than the typical interval between previous injections.

### Data collection

At each visit (day 180 and 365), pet owners completed an electronic PVAS assessment and investigators completed a CADESI-4 [23] score and an investigator skin lesion visual analog assessment (VetVAS). All lokivetmab injections were captured in an administration log for each dog and any medication administered during the period was recorded. All dogs were examined by the same site investigator on days 180 and 365.

#### PVAS

Dog owners electronically captured the severity of the pruritus using a PVAS score consisting of a 100 mm line with pruritus descriptions at 20 mm intervals [11]. A score of ≤ 20 mm would be consistent with a normal dog. Dogs who entered the initial study started with a score of ≥ 50 mm which would represent a dog with moderate pruritus [25]. A score of 100 mm would represent a dog with extremely severe pruritus. Owners were asked to complete the PVAS assessments electronically every 14 days.

##### VetVAS

The investigator VetVAS (reported in mm) is a subjective investigator assessment used to semi-quantitatively score the extent and severity of skin lesions and has been used in several dermatological studies [3, 18, 19]. For this study, Investigators were instructed to mark a single horizontal line on the vertical scale to record the severity of the dog’s skin condition for each visit. Normal skin would be scored as 0 with extreme dermatitis scored as 85 mm. A score of ≤ 15 mm aligned with mild dermatitis.

#### CADESI-04

The CADESI-04 was completed by the investigator at each visit. For the CADESI-04, 20 body regions were scored from 0 to 3 for 3 different lesion types for a possible maximum score of 180. A total score of ≥ 60 represents severe CAD, a score of 35–59 represents moderate disease, while mild skin lesions would be scored between 10–34. A score of < 10 would represent a CAD dog in remission (consistent with a normal dog) [23].

##### Pet owner experience

At each visit, pet owners were asked to complete a treatment satisfaction survey using a paper survey. Questions used a Likert scale and focused on product experience and overall satisfaction with the pet’s response to lokivetmab (Cytopoint) (Fig. 3).

##### Determination of data reliability

To be included in the data analysis, three criteria had to be met:

1. received lokivetmab every 4–8 weeks during the entire study interval
2. had a day 180 and 365 PVAS assessment
3. remained in the study until day 365

### Study size determination

Since this exploratory analysis utilized summary statistics, we relied on conventions from prior research as well as practical constraints to determine the number of study participants (at least 30 observations were needed for the Central Limit theorem to approximate normality).

### Data analysis

All variables were summarized but not statistically analyzed using SAS Proc Means (SAS 9.4, Cary, NC).

## Results

### Study population

Dogs (*n* = 95) from 8 clinics were initially enrolled in the study and 75 dogs successfully completed this study (Table 1). Six dogs were identified as having poor compliance or protocol deviations and either did not return within an 8-week period for another lokivetmab injection or did not return on Day 180 and/or Day 365. There were 7 dogs who withdrew due to episodes of dermatitis/otitis however, investigators did not provide additional information to document if these dogs withdrew because of pet owner decisions or based upon their treatment decision. Three dogs moved and were lost from follow-up and four dogs died during the course of the study; all 4 were senior dogs (over the age of 12 years) and the study investigators did not consider their death to be associated with treatment administration (Fig. 1).

**Table 1:** Reasons for Study withdraw

Reasons for study withdraw	# of dogs
Recurrent dermatitis/otitis	7
Moved/Died	7
Protocol deviation/poor compliance	6
Total	20

### Signalment

There were 36 male (48%) and 39 (52%) female dogs in this study. Mixed breed dogs represented 32 (43%) of total enrollment. Dogs ranged in age from 1 year of age to 13 yrs. old (average 5.9 yrs.) and weighed 2.5 to 59.5 kg (average 23.7 kg) (Tables 2, 3).

**Table 2:** Signalment

Age (yrs.)	Number of dogs (%)	Sex	Number of dogs (%)	Size	Number of dogs (%)
< 4	19 (25)	M	36 (48)	Toy/Small	23 (31)
4–8	36 (48)	F	39 (52)	Medium	20 (27)
> 8	20 (26)			Large	32 (42)
Unknown	1 (1)				
**Total**	75 (100)		75 (100)		75 (100)

**Table 3:** Breed

Breed	N
Mixed	32
Pit Bull	7
Golden Retriever	4
Boxer	4
Labrador Retriever	3
German Shepard	2
Other (1 per breed)	23

### Treatment administration

On Day 0 of the initial phase of the study, the actual dose of lokivetmab was 1.3–4.1 mg/kg (mean 2.5 mg/kg, median 2.4 mg/kg). The actual dose of lokivetmab during the continuation phase was 1.3–4.1 mg/kg (mean 2.6 mg/kg, median 2.5 mg/kg).

### Response to treatment

#### PVAS

**Fig. 2:**
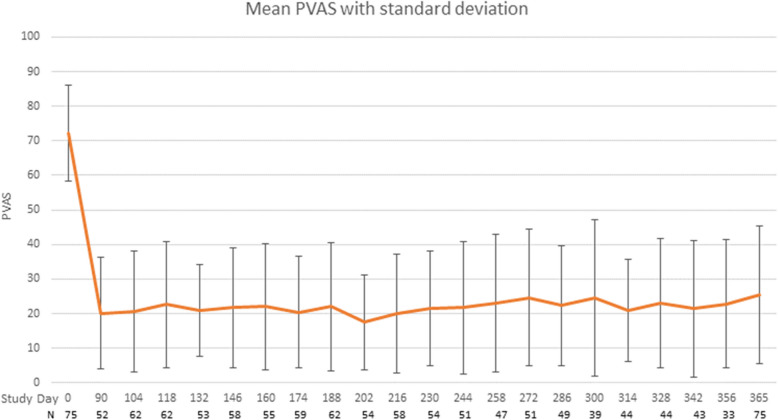
Mean PVAS with standard deviation

As the interval between dosing was individual patient dependent, dogs were evaluated on an individual dog basis to understand the overall efficacy of lokivetmab during the study. The overall mean PVAS for the study was 22.28 mm (range 0–50.1) (Table 4, Fig. 2).

**Table 4:** Mean PVAS and PVAS description (entire study duration)

Mean PVAS (range)	22 (0–50)
Number of dogs mean PVAS < 20 mm^a^	35
Number of dogs mean PVAS < 36 mm^b^	66
Number of dogs mean PVAS > 36 mm^b^	9

^a^ < 20 mm constitutes normal itch

^b^ICADA study guidelines

At the end of the study, there were 87% (64/75) of dogs who maintained a PVAS below their starting PVAS score on Day 0. Over the entire course of the study, 88% (65/75) of dogs obtained a mean PVAS below 36 mm and 47% (35/75) obtained a mean PVAS below 20mm (Table 4). Of those dogs, 31% (23/75) achieved a biweekly PVAS that was below 36 for the entirety of the study with 11% (8/75) having a biweekly PVAS score that stayed below 20 for the entire study (Table 4).

On day 180, 78% (59/75) achieved a 50% reduction in PVAS and 69% (52/75) on day 365 from their PVAS on day 0. Over the course of the 12 months, there were 20 dogs (20/75; 27%) whose PVAS stayed below 50% of their Day 0 PVAS during the entire course of the study.

#### VetVAS

The mean investigator VetVAS (reported in mm) was maintained below 15 throughout the entire study with a mean score of 14 on Day 180 and 13 on Day 365 (Table 5). The overall degree of dermatitis remained consistently low throughout the study with minimal numerical differences in VetVAS scores when comparing the dog’s entry into the continuation study (already receiving lokivetmab with controlled disease), to day 180 and day 365.

**Table 5:** Mean VetVAS score

Mean VetVAS score (mm)	Day 0^a^	Day 90^b^	Day 180	Day 365
Mean VetVAS (range)	24 (1–63)	12 (0–44)	14 (0–57)	13 (0–45)

^a^Day 0 was defined as the day of entry into the initial study

^b^Not every dog was evaluated on Day 90

#### CADESI-04

The mean CADESI-04 score on Day 0 was 38.3 (consistent with moderate skin lesions). The mean CADESI-04 score when dogs started this phase of the study was 17.7, showing a mean percentage improvement of 54% in the initial study phase. This improvement was maintained in the continuation phase reported here with a mean CADESI-04 score at Day 180 of 17.9 and 18.5 on Day 365 which is consistent with mild skin lesions. In addition, 67% (49/73; 2 dogs had missing data) of the dogs achieved a 50% or greater reduction of their CADESI-04 score at Day 180 and 68% (50/74; 1 dog had missing data) at Day 365. Overall, 52% (38/73) of dogs achieved and at each time point demonstrated a 50% CADEI-04 reduction over the course of the 12 months.

#### Recurrent infections

Over the course of the study, none of the dogs required any rescue treatment, however, 23 dogs developed a skin infection that required treatment. As noted above, seven (7) of these dogs did not complete the study, (Table 1) but 16 dogs (21%) were treated for their skin infection and completed the study. These 16 dogs experienced staphylococcal pyoderma, and/or *Malassezia* (yeast) dermatitis that involved the skin and/or ears and were treated for 31 infections (maximum of 3 infections over the course of the study in any single dog). Five dogs were treated once, 6 dogs were treated twice, and 5 dogs were treated 3 times as follows; systemic antimicrobials (12 times), topical antimicrobials (7 times) and a combination of topicals and systemic antimicrobials (6 times). In 6 treatment instances, the specific treatment was not provided. The mean PVAS for these dogs immediately prior to infection was 18 mm (5–39 mm), at the time of presentation when infection was present, the mean PVAS was 56 mm (range 37–84) and following 2 weeks of treatment for their skin infection, the mean PVAS returned to 21 mm (range 0–41).

#### Injection interval

Injections were administered on a 4–8 week basis based on the pet owner’s assessment of pruritus and investigators did not define the reinjection interval. This interval was not consistent for any dog throughout the study, thus, a dog could receive a minimum of 6 injections up to a maximum of 11 injections depending on the pet owner’s assessment of pruritus and willingness/ability to return to the clinic. Thirty-four (45%) of dogs had a mean interval of 4–5 weeks; 30 dogs (40%) had a mean interval of 6–7 weeks and 11 dogs (15%) had a mean interval of 7–8 weeks. The overall mean PVAS scores and ranges were similar regardless of the injection interval (Table 6). In additional the VetVAS and CADESI-04 scores were similar across these groups.

**Table 6:** Mean PVAS score by interval and number of Cytopoint injections (mm)

Interval	Score (range)	# of injections (N)	Score (range)
4–5 week interval	24 (8—34)	1 (54)	22 (0–49)
5–6 week interval	24 (1—50)	2 (14)	23 (8–40)
6–7 week interval	22 (2—49)	3 (7)	23 (2–50)
7–8 week interval	18 (0—47)		
Overall	22		

Because injections were administered based on a pet owners’ perception of pruritus, not every dog received a lokivetmab injection preemptively before a flare or escalation of pruritus occurred. Forty-three (43) dogs received a lokivetmab injection at their day 180 visit and forty-eight (48) dogs received an injection at their day 365 visit.

#### Pet owner questionnaire

At the conclusion of the study, pet owners were asked about their overall experience and satisfaction with lokivetmab (Cytopoint). Sixty-nine owners (92%) provided responses to the survey. When pet owners considered their satisfaction with lokivetmab, 93% (64/69) were satisfied/extremely satisfied with lokivetmab. When pet owners considered their dog’s reduction in allergic itch over time, 91% (63/69) felt their dog’s itch was reduced to a similar level always or most of the time. When asked about the duration of response to lokivetmab during the study, 9% (6/69) of owners reported their dog’s duration of response increased during the study period, while 77% (53/69) reported a similar duration always or most of the time.

At the end of the study, pet owners were asked to consider their pet’s skin condition when evaluating their dog’s QOL; 94% (65/69) reported feeling their dog’s QOL was good, very good or excellent. In addition, when considering how their dog’s skin condition impacted the owner QOL, 91% (63/69) reported their QOL was good or very good.

**Fig. 3:**
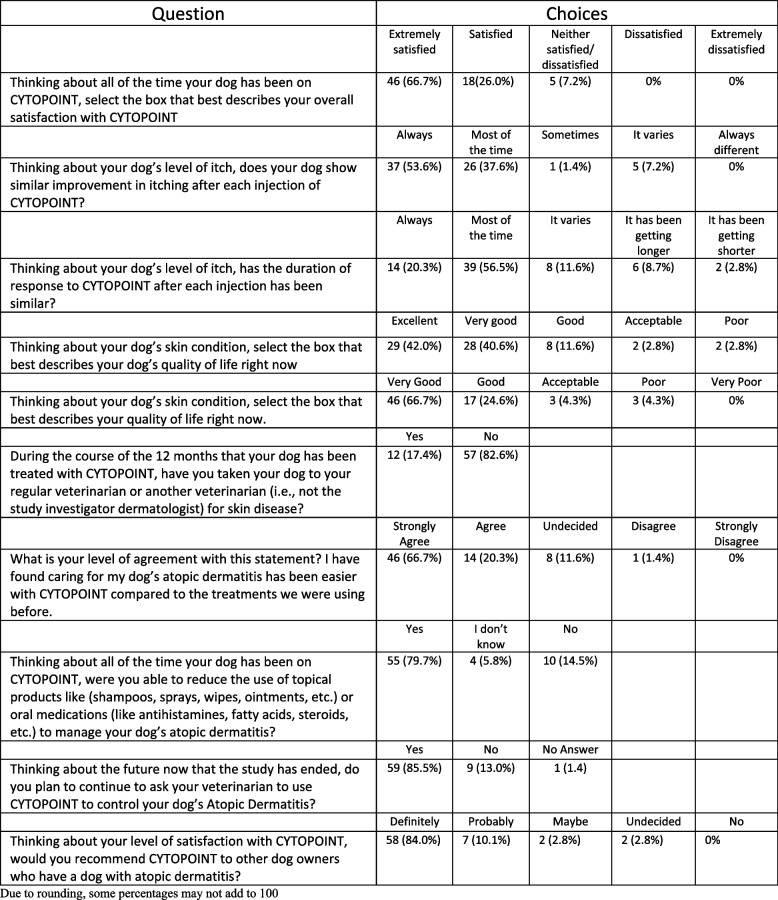
Pet owner satisfaction questionnaire

When considering how lokivetmab impacted their ability to care for their dog and provide treatment, 87% (60/69) agreed or strongly agreed that caring for their dog was easier when using lokivetmab as compared to prior treatment. Pet owners were permitted to use corticosteroid-free and antihistamine-free topicals (maximal use every other day) and shampoos (maximal use weekly) during the study and 80% (55/69) noted they were able to reduce the use of these products while receiving lokivetmab. At the end of the study, 86% (59/69) of owners planned to continue to use lokivetmab on their pet and 94% (65/69) of pet owners would recommend lokivetmab to other dog owners whose dogs suffer from atopic dermatitis (Fig. 3).

## Discussion

The goal of this study was to follow a cohort of dogs with CAD who received lokivetmab for an additional 9–11 months after they had achieved treatment success after an initial 1, 2 or 3 injections of lokivetmab, and characterize the ongoing benefit of this treatment. While the primary outcome, reduction in PVAS < 36 was selected based on the available COSCAD recommendations [22], previous research has suggested many (88% of 314 dogs) apparently healthy dogs have a PVAS ≤ 19 mm [25]; thus, the authors felt it relevant to include analysis of PVAS < 20 mm in the study outcomes (Table 4). In addition, this study was designed to include all dogs on a given therapy. Accordingly intention to treat analysis, which requires a control and treatment group for those dogs who either had poor compliance or protocol deviations, was by the very definition not available.

Given that chronic CAD requires lifelong treatment often with a combination of products to control pruritus and treat skin lesions [21], it is to be expected that a population of dogs in the study exhibited atopic flares when exposed to allergens during the course of the year. Recent studies have highlighted the importance of the “proactive approach when managing CAD” versus the “reactive approach” [16]. Ideally, when using lokivetmab, proactive management prior to a flare allows ongoing neutralization of IL-31 which targets the origin of the pruritic response [10, 16]. In this study, pet owners determined their own schedule for reinjection (within the 4–8 week parameters) based on initial duration of effect, which, at times was more about convenience or impacted by other family or personal factors. All of these dogs included in this study achieved a reduction in PVAS < 36 mm when receiving monthly lokivetmab, with 65% of dogs achieving a PVAS reduction < 36 mm after 1 injection, 85% (cumulative) after 2 injections and 93% (cumulative) after 3 injections of lokivetmab (Cytopoint) [9], which is consistent with initial pivotal studies [18]. However, when considering the PVAS ranges for the various intervals between lokivetmab injections (Table 6), the broad range of PVAS scores indicate there were some dogs who would have benefitted by having a shorter treatment interval. Indeed, based on increasing PVAS scores, over half of the dogs should have returned 1–2 weeks prior to their Day 180 (57%, 43/75) and Day 365 (64%, 48/75) visits for an injection but instead received an injection at the prescheduled visit. This represents a very real-world picture of CAD in practice and the importance convenience plays for pet owners and how easy it is to move away from proactive management of CAD. This also underscores the benefit of using lokivetmab in a prescriptive and preemptive manner for dogs diagnosed with CAD by forward booking the next lokivetmab injection before the pet owner leaves the practice.

In this study, lokivetmab was intended to act as an anchor therapy, with the majority of dogs utilizing treatment with lokivetmab alone and short-term supplementary topical or focal therapies only as needed. During the 12-month study, none of the dogs required rescue treatment with systemic products like Oclacitinib (Apoquel) or oral steroids. All of the dogs in this study achieved periods of pruritus reduction below a PVAS of 36, thus even dogs whose mean PVAS appear > 36 mm (12%; Table 4) received appreciable relief from the pruritus associated with their atopic disease. In addition, the reduction of CADESI-04 and VetVAS scores show a resolution of, and ongoing protection from, inflammation and lesions associated with pruritus over time.

Management of pruritic skin disease often presents challenges for both pet owners and veterinarians including increased caregiver burden [30]. Caregiver burden has been defined as a reaction of strain caused by providing care for a loved one with an illness and has been associated with depression and reduced quality of life in studies involving companion animal owners [29, 33]. Greater caregiver burden is associated with a lower quality of life and both caregiver burden and quality of life have a distinct link to a pet’s chronic disease [30].

Recent studies have shown the importance of starting with the simplest effective treatment when treating a dog with CAD, as it may reduce owner strain [31] and the transfer of caregiver burden onto veterinary staff [31]. When complex treatments can be reduced, clients view a veterinarian as more compassionate and trustworthy, thus improving the working relationship between a veterinarian and pet owner [32]. In addition, a pet owner’s belief that there is good control of chronic disease reduces caregiver burden [33].

In our study, pet owners reported the reduction of topical and oral medication usage during the study. This reduction in treatment led to pet owner’s reporting a “very good” quality of life (Fig. 3). Pet owners reported consistent duration and improvement in their pet’s CAD over the course of the study (Fig. 3) confirming their belief that lokivetmab was controlling their pet’s CAD and ultimately leading to an improved quality of life for the dog. As we impacted the pet owner’s view of their pet’s chronic disease through reduction of additional products and simplifying treatment options, we assisted in the reduction of the caregiver burden. At the same time, pet owners felt their pet’s CAD was well controlled, also aiding in the reduction of the pet owner’s caregiver burden. Prior work has described the relationship between high caregiver burden and low pet/pet owner QOL [30]. Thus decreasing the pet owner’s caregiver burden while increasing the overall wellbeing of the dog led to increased pet owner satisfaction.

CAD is a lifelong disease, therefore, setting reasonable pet owner expectations at the start and throughout the life of the pet is critical. Since skin disease may not be perceived as a “true” chronic disease, it is not uncommon for pet owners to expect complete resolution of a pet’s atopic disease after a single treatment. In this study, only dogs who responded to treatment with lokivetmab were included. For some dogs, 100% resolution of their clinical signs may not be possible with lokivetmab alone. The Fear Free movement has suggested setting pet owner expectations that controlling 80% of clinical signs is achievable for most dogs [6] and will help pet owners understand the importance of regular preemptive therapy with lokivetmab.

Our study design presents limitations in that neither the owners nor the veterinarians were blinded to the treatment group and we lacked the of comparison with a control placebo group or other antipruritic therapy. In addition, the variable nature of reinjection frequency allowed pet owners flexibility, but added a level of challenge in consistent management of pruritus. While dogs in this study had confirmed CAD, any flares in disease during the study did not require additional diagnostics through individual cases may have had further workup. Allergen identification through intradermal testing or serology was not required but may have been performed at the discretion of the Investigator. As allergen identification was not universally performed, this did not allow for other additional supportive treatments like allergen avoidance or lifestyle modification. Dogs whose pruritus was not as well controlled during this study may have benefited from this additional workup. Dog owners included in this study had dogs with a history of chronic dermatitis, thus their experience and treatment expectations may differ from other dog owners with acute or short-term allergic disease. Despite these limitations, because of the ease of administration and the lack of restrictions for age, breed, chronicity or type of allergy, this real world study provides support to include lokivetmab in the canine toolbox of choices to combat allergic skin disease.

## Conclusion

This study adds to the existing prospective evidence showing lokivetmab is effective when used for long term treatment of pruritus in dogs with CAD. While veterinarians should consider pet owners’ willingness and ability to treat their dog’s allergic disease when selecting a treatment protocol, our study has demonstrated lokivetmab is an effective foundational treatment which helps to minimize caregiver burden, improve the pet and owner quality of life, and ultimately support the pet-pet owner relationship.

## Data Availability

The datasets were obtained from a data sharing agreement and the data and analysis are the proprietary property of Zoetis, LLC.
